# Natural Bioactive Compound-Integrated Nanomaterials for Diabetic Wound Healing: Synergistic Effects, Multifunctional Designs, and Challenges

**DOI:** 10.3390/molecules30122562

**Published:** 2025-06-12

**Authors:** Tao Lu, Xuan Zhou, Shuai-Yu Jiang, Qing-Ao Zhao, Zi-Yi Liu, Dao-Fang Ding

**Affiliations:** 1 School of Traditional Chinese Medicine, Shanxi Datong University, Datong 037009, China; 03220010@sxdtdx.edu.cn (T.L.); jiangsy@sxdtdx.edu.cn (S.-Y.J.); 231005011136@sxdtdx.edu.cn (Q.-A.Z.); 2 Datong Key Laboratory of Smart Medicine and Health Care for Elderly Chronic Diseases, Shanxi Datong University, Datong 037009, China; 3 Institute of Rehabilitation Science, Shanghai University of Traditional Chinese Medicine, Shanghai 201203, China; xuanzhou0530@gmail.com

**Keywords:** nanomaterials, diabetic wound, natural products, molecular, mechanism

## Abstract

Diabetic wounds, as one of the most challenging complications of diabetes, exhibit impaired healing due to hyperglycemia, infection, vascular damage, microvascular deficits, dysregulated immune responses, and neuropathy. Conventional treatments are often limited by low drug bioavailability, transient therapeutic effects, and insufficient synergy across multiple pathways. Natural bioactive compounds are potential alternatives due to their multifunctional properties, including antioxidant, antimicrobial, and proangiogenic activities; however, their application is constrained by poor water solubility and rapid metabolism. Their integration with natural or synthetic nanovehicles significantly enhances stability, targeting, and controlled-release capabilities, while enhancing synergistic antimicrobial, immunomodulatory, and pro-repair functions. This review systematically catalogs the application of nanomaterial-loaded biomolecules, focuses on innovative progress in plant-based and animal-derived nanosystems, and further elucidates the multimodal therapeutic potential of synthetic–natural hybrid nanosystems. By synthesizing cutting-edge research, we also summarize advantageous features, development prospects, and existing challenges from the three dimensions of mechanistic evidence, preclinical validation, and current nanodelivery platforms, and propose a framework for grading application potential to provide a theoretical basis and strategic guidance for the rational design and clinical translation of future nanomedicines.

## 1. Introduction

Diabetes is one of the most common chronic diseases of our time. An estimated 828 million adults have diabetes in 2022, increasing from 198 million in 1990, according to a 2024 study [1]. Among these individuals, approximately 25% experience diabetic wound healing issues, a percentage that continues to rise as the disease progresses [2]. 

**Figure 1:**
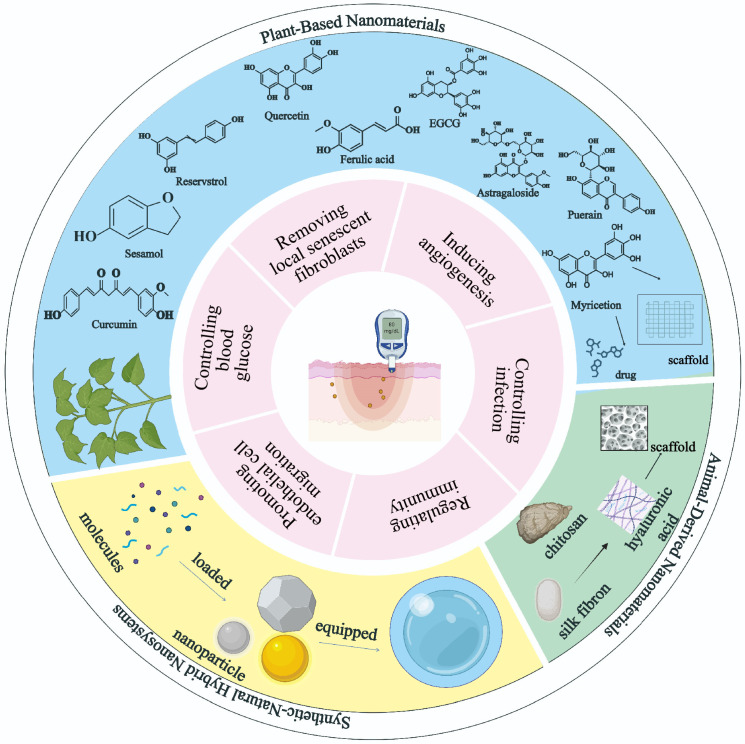
Overview of the article. This figure demonstrates the versatile application of nanomaterials in diabetic wound therapy. At the center is a representation of a diabetic skin lesion. Surrounding it is a two-layered structure: the inner layer highlights six distinct repair mechanisms in diabetic wound healing mediated by nanomaterials, while the outer layer is divided into three main categories: plant-based nanomaterials (blue area), animal-derived nanomaterials (green area), and synthetic natural hybrid nanosystems (yellow area).

The refractory nature of diabetic wounds stems from a multifactorial etiology, including (1) persistent hyperglycemia, (2) chronic infection, (3) vascular damage and microvascular deficits, (4) dysregulated immune responses, and (5) neuropathy (Figure 1). These factors collectively contribute to hypoxia, necrotic tissue accumulation, persistent inflammation, and anaerobic infections, further aggravated by hyperglycemia-driven bacterial proliferation and impaired oxygen delivery [3,4]. Current treatment strategies focus on blood glucose control, infection management, immune regulation, microcirculation improvement, epithelial regeneration, etc. However, the pathophysiological complexity of diabetic wounds and the limitations of conventional therapies (e.g., wound dressings and surgical debridement) often lead to suboptimal healing outcomes owing to insufficient drug bioavailability, transient therapeutic effects, and limited capacity to address multiple pathological pathways concurrently [5]. In contrast, natural products hold significant promise due to their intrinsic multifunctionality, which enables modulation of various pathogenic mechanisms implicated in diabetic wound healing.

Moreover, the integration of nanomaterials with these bioactive compounds opens new avenues in the management of diabetic wound healing. This combinatorial approach not only enhances target specificity but also offers a safer and more biocompatible alternative to traditional therapies. The convergence of nanotechnology and natural compound-based therapy represents a transformative advancement in the development of innovative, multi-targeted treatment strategies for diabetic wounds, with the potential to significantly improve healing outcomes and patient quality of life [6]. 

However, critical research gaps need to be addressed to increase the therapeutic potential of natural product-based nanomedicine for diabetic wound healing. A significant challenge lies in the limited understanding of molecular mechanisms underlying the synergistic tissue-regenerative effects of bioactive compounds and nanomaterials. Further research is also needed to optimize nanocarrier systems for the controlled and targeted delivery of these agents. Although nanomaterials offer a promising platform for enhancing drug bioavailability, stability, and therapeutic efficacy, the design of such systems requires refinement to ensure precise localization, sustained release, and biocompatibility. Recent advances in smart hydrogels and functionalized nanoparticles (NPs) have demonstrated significant potential in preclinical models of diabetic wound care [7]. Nevertheless, the clinical translation of these findings remains limited, primarily due to the lack of sufficient long-term safety data and a shortage of large-scale, well-controlled trials. Therefore, comprehensive investigations are needed to evaluate both the safety profiles and therapeutic efficacy of these integrated nano-systems in real-world trials [8]. Addressing these research gaps will require a multidisciplinary approach that combines insights from materials science, pharmacology, and clinical medicine. By learning more about the latest research advances in the interaction between natural bioactive compounds and nanomaterials, the development of more effective therapies for diabetic wound healing will ultimately improve patient outcomes and quality of life. To this end, this review provides a comprehensive analysis of the latest progress in the application of nanomedicine-based natural products for diabetic wound healing. We systematically examine their therapeutic efficacy, mechanisms of action, and translational potential. Nanomedicine has emerged as a powerful platform in this context, offering key advantages such as high drug-loading capacity, biocompatibility, tunable surface chemistry, and the ability to co-deliver multiple agents with synergistic functions, including antimicrobial effects, anti-inflammatory properties, and enhanced cell proliferation. Despite these promising advances, clinical studies evaluating such integrative strategies remain scarce. Consequently, this review innovatively integrates synergistic effects among naturally derived nanomaterials, natural bioactive molecules, and synthetic nanomaterials to facilitate the translation of preclinical innovations into personalized diabetic wound therapies.

## 2. Natural Product-Derived Nanomaterial

Nanotechnology-based scaffolds have emerged as transformative platforms in the management of diabetic wounds. Capitalizing on high surface area-to-volume ratios and tunable porosity, these materials closely mimic the extracellular matrix (ECM) architecture while enabling controlled drug release [9,10] (Table 1). These three-dimensional systems offer dual functionality as both structural supports and bioactive delivery vehicles, with plant-derived polysaccharides, such as alginate, demonstrating exceptional moisture retention and intrinsic antimicrobial activity [11,12]. Concurrently, animal-derived biopolymers such as collagen and silk fibroin (SF) offer evolutionarily optimized microenvironments [13,14].

### 2.1. Plant-Based Nanomaterials

#### 2.1.1. Plant-Extracted Molecule Compound Loaded in Nanoplatforms

As the multi-targeted roles of natural products in diabetic wound repair are gradually revealed, their incorporation into nanodelivery systems has received increasing attention. This chapter provides a comprehensive overview of nine extensively studied natural bioactive compounds employed in nanomedicine for the treatment of diabetic wounds. Table 2 summarizes these compounds incorporated into nano-delivery systems for diabetic wound healing, comparing their vehicle compositions, disease models, and therapeutic mechanisms. 

##### Curcumin

Curcumin, the main bioactive component in turmeric, possesses antioxidant, antibacterial, anti-inflammatory, and angiogenesis-promoting properties [28]. These multifaceted therapeutic effects make curcumin a promising candidate for enhancing tissue repair, especially in chronic diabetic wounds, where persistent inflammation and oxidative stress impair the healing process. However, the application of curcumin is limited by its poor solubility and low oral bioavailability. To overcome these limitations, curcumin has been incorporated into various nanodelivery systems to enhance its stability, bioavailability, and targeted efficacy [29]. For instance, chitosan NPs-encapsulated curcumin (CUR-CSNPs) have demonstrated improved solubility and stability, which can also reduce macrophage-mediated inflammation, promote angiogenesis, and exhibit significant wound healing activity, along with notable antioxidant and cell proliferation effects [30,31]. 

##### Sesamol

Sesamol, a natural antioxidant derived from sesame, exhibits potent free radical scavenging, anti-inflammatory, and antifungal properties [32,33], with demonstrated efficacy in diabetic wound healing [34]. However, the oral bioavailability of sesamol is only 35%, and its conjugated metabolites are rapidly cleared within 0–4 hours post-administration [35,36]. To address this limitation, researchers have incorporated sesamol into nanodelivery systems. Sesamol–poly(lactic-co-glycolic acid) (PLGA) nanosuspension has been reported to accelerate impaired trauma repair in diabetic foot ulcers by prolonging the drug’s half-life and reducing blood glucose levels [37]. Similarly, sesamol-loaded composite nanofiber membranes have been shown to promote wound healing in diabetic mice by upregulating interleukin-6 (IL-6) expression, thereby promoting keratinocyte growth and proliferation [38]. Recently, a novel topical formulation utilizing solid lipid nanoparticles (SLNs) for sesamol delivery has expanded its therapeutic potential in diabetic wounds, promoting tissue regeneration by enhancing skin retention, antimicrobial activity, and modulation of oxidative stress [39]. 

##### Quercetin

Quercetin facilitates wound healing by activating the Wnt/β-catenin signaling pathway [40]. Moreover, its ability to suppress the NF-κB signaling pathway plays a crucial role in modulating the inflammatory response, promoting wound healing, and the remodeling process [41]. However, due to its poor water solubility as a lipophilic molecule, the efficient delivery of quercetin requires the use of appropriate carrier systems [42]. Nanocrystals and polymeric NPs have been shown to enhance their bioavailability and increase skin penetration [43]. Notably, quarternized CS/quercetin/polyacrylamide semi-interpenetrating network hydrogel has demonstrated potent antibacterial properties, structural robustness, and self-recovery capacity, making it well-suited for wound healing applications [44]. Furthermore, water-soluble quercetin–borate NPs have been developed to form antimicrobial quercetin–borate–PVA composite microgels, which are used to produce xerogel films, significantly accelerating the healing of full-thickness skin wounds [45]. Another innovative strategy involves a collagen–nanomaterial–drug hybrid scaffold based on a graphene oxide–polyethylene glycol-quercetin complex (GO-PEG/quercetin) integrated into an acellular dermal matrix (ADM-GO-PEG/quercetin), which further promotes collagen deposition and angiogenesis in diabetic wound repair [46].

##### Ferulic Acid

Ferulic acid has demonstrated antioxidant and antidiabetic properties. However, the poor solubility and bioavailability of ferulic acid significantly hinder its biomedical applications. Both NPs and nanofibers have proven their benefits as drug carriers [47]. Ferulic acid-poly(lactic-co-glycolic acid) (ferulic acid-PLGA) NPs accelerated wound healing in diabetic rats and increased hydroxyproline content significantly [48]. In addition, ferulic acid-loaded nanofibers exhibit notable antimicrobial activity and demonstrate strong efficacy in accelerating wound healing in diabetic models [49]. Furthermore, an innovative nanoemulgel formulation has also been developed to improve the intracellular delivery of ferulic acid, expanding its therapeutic potential in diabetic wound management [50].

##### Epigallocatechin Gallate (EGCG)

EGCG-based nanoformulations exhibit multifaceted therapeutic potential in the management of diabetic wounds. For instance, a composite formulation comprising EGCG, gelatin, ascorbic acid, and CSNPs was prepared via an ion cross-linking method. This nanoformulation promotes diabetic wound healing by enhancing collagen deposition, stimulating angiogenesis, and reducing inflammatory cell infiltration [51]. Similarly, a nanoliposomal formulation of EGCG has been shown to enhance plasma antioxidant capacity in streptozotocin-induced diabetic rats [52]. Furthermore, green tea polyphenol nanospheres encapsulated in PVA/alginate hydrogel matrix demonstrate superior efficacy compared to non-nanosphere formulations in mitigating inflammation and accelerating the healing of diabetic skin wounds by activating the PI3K/AKT signaling pathway [53].

A recent study investigating the combination of EGCG and berberine (BBR) demonstrated potent synergistic antibacterial effects against multidrug-resistant bacterial strains [54]. Currently, polyphenol substances similar to EGCG are also gaining significant attention. Protocatechuic aldehyde-based microneedle patch, a polyphenol–metal nanocomposite system that offers pH-responsive drug release and activates the insulin signaling pathway, eliminating bacterial biofilms, promoting macrophages polarization towards the anti-inflammatory M2 phenotype, scavenging reactive oxygen species (ROS), and comprehensively modulating the diabetic wound microenvironment [55].

##### Astragaloside

Astragaloside, a bioactive ingredient extracted from Astragalus membranaceus, possesses anti-inflammatory, immunomodulatory, and anti-fibrotic properties. In diabetic rat models, wound healing is significantly enhanced when astragaloside is encapsulated with matrix metalloproteinase(MMP)-2-responsive hyaluronic acid (HA)-conjugated polyamidoamine dendrimers [56]. In addition, astragaloside has been shown to promote vascularization and collagen deposition, enhance epithelial regeneration, and facilitate endothelial cell migration and lumen formation through the activation of the SUMOylation pathway [57]. Additionally, astragaloside IV can increase insulin levels and exert antioxidant capacity by activating the JNK/Nrf2 pathway, suggesting a potential therapeutic role in wound healing for type 1 diabetes [58,59].

##### Resveratrol (RES)

Resveratrol, an active compound derived from *Vitis vinifera*, has been shown to reduce insulin resistance and promote diabetic wound healing by activating the SIRT1-FOXO1-C-Myc pathway [60,61]. Incorporation of resveratrol into advanced nanoplatforms can enhance functional versatility. For example, gelatin methacrylate (GelMA)/SF glycidyl methacrylate (SFMA)/platelet-derived extracellular vesicles (PDEVs)/mesoporous silica NPs loaded with resveratrol (GelMA/SFMA/PDEVs/MSN-resveratrol) hydrogels, which upregulated the expression of Arg-1 and transforming growth factor-β1 (TGF-β1), downregulated TNF-α and iNOS, and promoted angiogenesis, thereby accelerating wound healing in diabetic mice [62].

##### Puerarin (PUE)

Puerarin, a bioactive compound derived from *Pueraria montana* var. *lobata* (Ohwi) Maesen & S. M. Almeida [63], has been shown to promote angiogenesis [64]. However, its application is limited by poor water solubility and low bioavailability [65]. To address these challenges, Su et al. [66] developed L-arginine-modified puerarin carbon NPs, which retain the cell proliferation and angiogenesis properties while also imparting synergistic antimicrobial functions. This nanomaterial effectively reduced inflammatory infiltration and initiated functional vascular regeneration in diabetic rat wounds within 3 days post-intervention. Furthermore, CS@PUE (puerarin) nanofiber hydrogels demonstrated the ability to inhibit the ectopic elevation of miR-29a/b1 in wounds of type I diabetic mice, thereby promoting vascular neovascularization and collagen synthesis, ultimately accelerating diabetic wound healing [67].

##### Myricetin 

Myricetin, a flavanone compound originally isolated from the bark and leaves of Myrica rubra (Morella rubra Lour.), alleviates type 2 diabetes in mice by inhibiting sugar digestion and absorption, as well as modulating intestinal flora [68,69]. To address targeted delivery challenges, researchers developed a novel composite hydrogel system [70]. In this system, glucosensitive phenylboronic acid groups were first grafted onto HA chains through a one-step synthesis, followed by integration into a poly (ethylene glycol) diacrylate hydrogel matrix. The polyphenol moiety of myricetin binds to the hybrid hydrogel through the formation of dynamic borate bonds with the phenylboronic acid groups. Under the high-glucose microenvironment of chronic diabetic wounds, this hydrogel can responsively release myricetin, effectively scavenging ROS, improving the inflammatory response, and accelerating angiogenesis, ultimately promoting diabetic wound healing in rats.

The natural compounds were systematically categorized into high, medium, and low priority tiers based on the following criteria: preclinical validation (including in vitro and in vivo experiments), the number and quality of publications, the depth of elucidated molecular mechanisms, and compound toxicity, clearly highlighted in Table 3.

Curcumin (high priority) demonstrates multifunctional antioxidant, antibacterial, and pro-angiogenic effects, with CS-based nanoformulations enhancing bioavailability and accelerating wound closure [29]. However, long-term biocompatibility evaluations are needed [31]. Sesamol (high priority) promotes diabetic ulcer healing via PLGA-based nanosuspensions, which prolong drug retention and upregulate IL-6-driven keratinocyte proliferation; however, clinical validation and pharmacokinetic optimization remain limited [37,38]. EGCG (high priority), particularly when combined with berberine (BBR), exhibits strong antimicrobial activity against multidrug-resistant bacteria, while also promoting collagen deposition through extracellular vesicles and nanoliposomal systems. Yet, stability concerns persist with non-encapsulated EGCG [51,52,53]. These three natural compounds represent top-tier therapeutic candidates for diabetic wound management, supported by robust preclinical data, optimized nanodelivery platforms, and mechanisms that holistically address the pathophysiology of diabetic wounds. Quercetin (medium priority) activates the Wnt/β-catenin signaling pathway to resolve inflammation, and its therapeutic efficacy depends on the optimization of nanocrystal or hydrogel-based delivery systems [40,43,44]. Ferulic acid (medium priority) enhances hydroxyproline content and improves wound closure via PLGA NPs; however, its validation in complicated wound infections remains lacking [48,49]. Astragaloside IV (medium priority) modulates oxidative stress via matrix metalloproteinase-2-responsive dendrimers and activates JNK/Nrf2 pathways [56,58,59]. Resveratrol (medium priority) promotes SIRT1-mediated angiogenesis when incorporated into hydrogel systems, but it faces challenges related to rapid metabolism [60,62]. These compounds exhibit therapeutic potential but require further optimization in formulation design or deeper mechanistic validation to enable successful clinical translation.

Puerarin (low priority) promotes vascularization in type 1 diabetic mice through an injectable chitosan-puerarin nanofiber hydrogel. This system accelerates wound healing by suppressing the expression of miR-29a/b1 [67]. Myricetin (low priority) is delivered via glucose-responsive hydrogels that dynamically scavenge ROS and stimulate angiogenesis; however, it lacks comparative studies against existing therapies [70]. Overall, these compounds have limited or early-stage evidence for diabetic wound healing and face notable translational challenges, rendering them a low priority for further development at present.

**Table 2:** Small Molecule Compounds in Diabetic Wound Healing.

Small Molecule Compound	Vehicle	Components	Model	Mechanisms	Ref.
Curcumin	CSNPs	CS; Aqueous acetic acid; Sodium; Tripolyphosphate; Anhydrous ethanol; Curcumin.	Diabetic rat excision skin wound model	Regulating immunity Inducing angiogenesis	[31]
Sesamol	Cellulose acetate (CA)–zein composite nanofiber membrane	Acetic acid; Zein; Sesamol.	Diabetic mice excision skin wound model	Regulating immunity Enhancing keratinocyte proliferation and migration.	[38]
Quercetin	ADM-GO-PEG hybrid scaffold	Graphene oxide(GO) sheet; 6-armed PEG; Quercetin; ADM.	Diabetic mouse excision skin wound model	Inducing angiogenesis	[46]
Ferulic acid	Silk-sericin-based CA and PCL hybrid nanofibers	CA; PCL; Acetone; Ferulic acid; Silk sericin protein.	Diabetic rat full-thickness skin wound model	Regulating immunity Promoting endothelial cell migration.	[49]
Epigallocatechin gallate	Ascorbic acid, gelatin, and CSNPs	CS; Gelatin; Sodium EGCG; Ascorbic acid.	Diabetic mouse full-thickness skin wound model	Regulating immunity Inducing angiogenesis	[51]
Astragaloside	Matrix metalloproteinase-2-responsive HA end-conjugated polyamidoamine(HA-pep-PAMAM) dendrimers	HA; PAMAM; Astragaloside IV.	Diabetic mouse excision skin wound model	Regulating immunity	[56]
Resveratrol	GelMA/SFMA/PDEVs composite hydrogel	Gelatin; Methacrylic anhydride SF; Glycidyl Methacrylate; Amino-functionalized MSN NPs; Resveratrol; PDEVS; LiBr; Phenyl-2,4,6trimethylbenzoylphosphinate.	Full-thickness skin defect model of diabetic mice	Regulating immunity Inducing angiogenesis	[62]
Puerarin	CS nanofiber hydrogel	CS; Puerarin.	Type I diabetic mouse model of full-thickness skin wound	Regulating immunity Inducing angiogenesis	[67]
Myricetin	PEG–acryloyl chloride/phenylboronic acid–HA hybrid hydrogel	PEG; Acryloyl chloride; HA, myricetin.	Type I diabetic rat model of full-thickness wound	Regulating immunity Inducing angiogenesis	[70]

#### 2.1.2. Plant Polysaccharide-Based Nanoscaffolds

##### Alginate

Alginate, a hydrophilic biopolymer, has been extensively studied for its application in wet wound dressings due to its excellent moisture-retention and biocompatibility properties [71]. When combined with other polymers, alginate-based nanopolymers can be engineered to modulate mechanical strength, gelation behavior, and cellular affinity, thereby enhancing their therapeutic utility [72]. For instance, Akbar et al. developed a curcumin-loaded polymeric micelle composed of CS, alginate, maltodextrin, and pluronic, which showed hypoglycemic effects in bisphenol A-induced diabetic rats and promoted wound healing in vivo [73]. Furthermore, an innovative NPs combining CS, hydroxypropyl methylcellulose, lidocaine chloride, sodium alginate, and mucin B sulfate (an antibiotic) has shown promising results in wound repair and regeneration in animal models [74]. CS-based biodegradable polymeric hydrogels containing silver nanoparticles(AgNPs) and calcium alginate NPs (Ca-AlgNPs) effectively enhanced diabetic wound healing through broad-spectrum antimicrobial activity, promoting the local accumulation of growth factors, platelets, circulating fibroblasts, and cytokines [75]. 

##### Cellulose

Cellulose, the most abundant organic polymer on Earth, serves as a structural polysaccharide in the cell walls of plants. Its derivatives (e.g., cellulose acetate and carboxymethyl cellulose) have emerged as versatile platforms for engineering advanced medical nanomaterials, particularly in addressing the complex pathophysiology of diabetic wounds [76]. Recent innovations highlight two distinct cellulose-based strategies. Carboxymethyl cellulose, engineered into thermos-responsive hydrogels with CS and Pluronic F127, enables sustained curcumin release, extending its half-life to 5.92 ± 0.7 h and enhancing wound contraction in diabetic rats by promoting collagen deposition and re-epithelialization [77]. In parallel, cellulose acetate nanofibers electrospun with polycaprolactone form a biomimetic scaffold that, when functionalized with CS-encapsulated cerium oxide NPs, achieves 89.59% antioxidant activity and 90.3% fibroblast migration within 15 days via ROS scavenging [78]. These cellulose-based nanocomposites leverage the polymer’s biocompatibility and structural adaptability, incorporating functional NPs to address key challenges in diabetic wound healing, including microbial resistance, oxidative stress, and impaired tissue regeneration.

**Table 3:** Nanocarrier-Mediated Delivery of Natural Bioactive Compounds for Diabetic Wound Healing.

Natural Bioactive Compounds	Functions	Perspectives	Limitations	Priority	Ref.
Curcumin	Strong antioxidant and anti-inflammatory effects.	Nanoformulations show potentials for targeted delivery and enhanced stability.	Poor water solubility. Low intrinsic bioavailability.	High	[28,29,31]
Sesamol	Powerful antioxidant and anti-inflammatory effects. Promoting IL-6-mediated keratinocyte proliferation.	Nanomaterials extend local retention time.	Limited clinical validation. Short biological half-life in free forms.	High	[32,33,35,36,37,38]
Quercetin	Anti-inflammatory effect via Wnt/β-catenin pathway and NF-κB pathway. Stimulating collagen deposition.	Optimized nanocrystal and hydrogel systems increase efficacy.	Poor solubility and bioavailability. Carrier-dependent efficacy.	Medium	[40,41,42,43,44,46]
Ferulic acid	Antidiabetic, antioxidant, and antimicrobial effects.	Nanoparticle encapsulation show enhanced stability. Emerging potential for controlling macrophage polarization.	Limited solubility and bioavailability.	Medium	[47,48,49,50]
Epigallocatechin gallate	Strong antioxidant activity. Stimulating collagen deposition.	Potential for combinatorial regimens.	Poor stability without encapsulation.	High	[51,52,53]
Astragaloside	Antioxidant and anti-inflammatory effects. Promoting angiogenesis and collagen deposition. Activating the JNK/Nrf2 signaling pathway to reduce oxidative damage.	Potential to combine with other materials for targeted delivery.	Bioavailability challenges.	Medium	[56,58,59]
Resveratrol	Strong antioxidant, anti-inflammatory effects. Reducing insulin resistance Upregulating SIRT1 to enhance angiogenesis.	Novel hydrogel/nanoparticle systems improve retention and efficacy.	Rapid metabolism. Limited stability in vivo.	Medium	[60,61,62]
Puerarin	Enhancing microcirculation and angiogenesis.	Advanced nanoplatforms. Potential therapeutic candidate for ischemic wounds.	Poor water solubility and bioavailability.	Low	[64,65,66]
Myricetin	Highly efficient ROS scavenging. Inhibiting the digestion and absorption of carbohydrate.	Promising, smart, glucose-responsive delivery. Emerging candidate for diabetic wounds.	Insufficient comparative studies. Limited long-term safety data.	Low	[68,69,70]
Alginate	Excellent hydrophilicity suitable for wet wound environments. Blending with collagen to enhance mechanical strength.	Strong potential for multifunctional polymeric micelle formulations. Smart dressings integrating antimicrobial peptides.	Clinical translation requires precise optimization of formulation variables to maintain functional integrity.	High	[72,74,75]
Cellulose	Providing structural support and exudate management via controllable nanofiber networks. Sustained therapeutic release (e.g., prolonged curcumin half-life) via thermosensitive hydrogels.	Promising potential in thermoresponsive hydrogels and advanced nanofiber scaffolds. Effective integration with functional NPs for enhanced multifunctional wound therapy.	Need strict optimization. Performance is highly dependent on the type of NPs.	High	[76,77,78]

### 2.2. Animal-Derived Nanomaterials

Alginate, collagen, CS, HA, and other animal-derived polymers have been extensively explored for their structural and functional resemblance to native ECM. When fabricated into nanomaterials, these biomolecules mimic the architecture of normal tissue while enhancing wound healing through facilitated cell migration, angiogenesis, and immunomodulation [79,80]. Table 4 summarizes the properties of these nanostructures, comparing their therapeutic advantages and limitations in diabetic wound healing.

#### 2.2.1. Collagen-Based Nanostructures

Collagen, one of the most extensively studied and utilized biomaterials, is a key component of the ECM. Its biodegradability, widespread availability, and appropriate mechanical strength render it highly suitable for innovative drug delivery systems, including nanoscaffolds and smart hydrogels [81,82]. Due to its inherent biocompatibility and structural characteristics, collagen provides a three-dimensional support matrix for micro- and nanoscaffolds, thereby facilitating cell migration and promoting neovascularization [83]. Moreover, bio-hybrid hydrogels incorporating collagen-capped AgNPs have been shown to mitigate oxidative stress within the wound microenvironment, thereby improving the inflammatory state [84]. In terms of dosage form innovation, the injectable collagen hydrogel enhances the mechanical properties of collagen and retains its native biocompatibility. This advancement addresses the limitations of conventional wound dressings in terms of adaptability and functionality, enabling effective treatment of deeper tissue injuries. Furthermore, these hydrogels exhibit excellent conformability to irregular wound shapes, with in vivo studies demonstrating a wound healing rate exceeding 80% by day 7 [85].

#### 2.2.2. Chitosan-Based Nanostructures

Chitosan, a widely used polymeric nanocarrier for wound healing, offers low immunogenicity, biodegradability, and excellent biocompatibility [86,87]. Calcium alginate hydrogel loaded with CSNPs exhibits multifunctional effects that accelerate wound healing: (1) exerting antibacterial activity; (2) stimulating interleukin-6 (IL-6) production and release in vascular endothelial cells; (3) facilitating vascular endothelial cell invasion, migration, and neovascularization [88]. In addition, even without drug loading, nanosystems formed by electrostatic complexation between positively charged CS and negatively charged alginate have demonstrated promising effects in treating both diabetic and non-diabetic pressure ulcers by their dual functions of structural support and immunomodulation, indicating that the intrinsic bioactivities of these polysaccharides themselves can create a wound environment conducive to healing [89]. When antioxidants are incorporated into CS-based carriers, their therapeutic efficacy is significantly enhanced. For example, rosmarinic acid-loaded CS-encapsulated graphene NPs formulation not only promotes epithelial reconstruction but also reduces post-healing scar formation [90]. Similarly, the synergistic combination of curcumin, CS, and collagen has emerged as a promising approach to address the multifaceted clinical challenges of diabetic wounds, resulting in more efficient wound healing [30]. Collectively, these CS-based nanomaterials provide broad-spectrum antibacterial protection, attenuate chronic inflammation and oxidative stress, and actively modulate immune response by shifting macrophages from a pro-inflammatory M1 phenotype towards a pro-regenerative M2 phenotype [91,92]. In addition, at the molecular level, these hydrogels can inhibit the excessive production of pro-inflammatory factors, such as IL-6, while upregulating anti-inflammatory factors, including IL-4 and TGF-β1, which can further promote ECM formation, neovascularization, and epidermal reconstruction [91,92].

#### 2.2.3. Hyaluronic Acid-Based Nanostructures

As a key component of the dermal ECM, HA plays crucial roles in modulating inflammation, promoting neovascularization, and facilitating tissue repair processes [93]. Leveraging these biological functions, researchers have developed HA-based nanomaterials to address the impaired healing observed in diabetic wounds. For instance, polysaccharide-decorated NPs loaded with vitamin E have been incorporated into polymeric films containing aloe vera extract, HA, sodium alginate, polyethylene oxide, and PVA, offering an innovative treatment for skin wounds [94]. In this multifunctional film, HA contributes to the formation of a moist, ECM-like environment with the potential to promote cell migration and angiogenesis. In diabetic models, a glucose-responsive antioxidant hydrogel platform based on HA has been shown to enhance collagen deposition and upregulate the expression of vascular endothelial growth factor (VEGF) and CD31 though lacking the ability to directly stimulate angiogenesis [95]. Beyond promoting matrix repair and angiogenesis, HA-based nanomaterials can modulate the immune microenvironment of chronic wounds. For example, HA-based hydrogel loaded with paeoniflorin has been shown to promote diabetic wound healing by inactivating STAT1 while activating STAT6 signaling, thereby polarizing macrophages towards the M2 phenotype [96]. Moreover, the versatility of HA as a nanocarrier enables the design of smart delivery systems that target the hostile microenvironment of diabetic wounds. For instance, Liu et al. [97] developed an HA-modified cascade nanosystem that targets bacterial infections in diabetic wounds. This system exploits both high-glucose conditions and the presence of endogenous hyaluronidase to degrade the HA shell, thereby releasing a cascade of nanoenzymes and natural lysozyme precisely at the site of infection. It further incorporates photothermal therapy and chemodynamic therapy to enhance antibacterial effects and accelerate wound healing, demonstrating the versatility of HA in advanced wound care.

#### 2.2.4. Silk Fibroin-Based Nanostructures

Silk fibroin, a major structural protein derived from silk, has emerged as a versatile biomaterial for diabetic wound management due to its excellent biocompatibility and biodegradable nature [98,99,100]. These characteristics enable its widespread application in fabricating nanostructured scaffolds, hydrogels, and drug-loaded NPs. Silk fibroin-based delivery systems exhibit precisely controlled drug release profiles, making them ideal carriers for delivering therapeutic agents to diabetic wounds [101,102]. To further enhance therapeutic efficacy, silk fibroin is often combined with complementary biomaterials. For example, its integration with polyglycolic acid imparts tunable biodegradability to the prepared scaffold, reducing the risk of secondary injury while creating space conducive to neovascularization during wound healing [103].

Beyond serving as a structural matrix, silk fibroin has a direct influence on cellular behavior. It has been shown to enhance the paracrine activity of stem cells, thereby modulating ECM deposition, promoting angiogenesis, and contributing to immunoregulation [104]. Recent advances in bioengineering have further expanded the potential of silk fibroin by enabling functional customization. Through genetic modification of silkworms, researchers have produced recombinant silk fibroin variants with intrinsic antibacterial properties, eliminating the need for exogenous drug loading and offering a novel, drug-free approach to infection control in wound healing [105].

**Table 4:** Common Characteristics of Animal-derived Natural Polymer-Based Nanostructures in diabetic wound healing.

Material	Therapeutic Advantages	Limitations	Ref.
Collagen-Based Nanostructures	Excellent biocompatibility and biodegradability. Supporting cell migration and vascularization. Adaptable to diverse dosage forms.	Suboptimal efficacy when used alone.	[81,82,85]
Chitosan-Based Nanostructures	Strong antibacterial activity against pathogens. High drug-loading capacity.	Suboptimal efficacy when used alone. Limited long-term biocompatibility data.	[79,86,90]
Hyaluronic Acid-Based Nanostructures	Regulating inflammation, angiogenesis, antibacterial activity and tissue regeneration.	Limited stimulation of endothelial cells and angiogenesis. Potential instability in complex formulations.	[93,94,95,96,97]
Silk Fibroin-Based Nanostructures	Superior biocompatibility and non-toxic degradation byproducts. Enabling precise drug release profiles for therapeutic delivery. Genetic modifiability for antimicrobial features.	Suboptimal structural properties when used alone. High costs for genetically engineered variants.	[98,99,100,101,102,103,104,105]

## 3. Synthetic–Natural Hybrid Nanosystems

### 3.1. Metallic NPs

#### 3.1.1. AgNPs

AgNPs, inheriting the ancient antimicrobial legacy of elemental silver, represent cornerstone nanomaterials in the management of diabetic wounds. Silver promotes skin wound recovery through multiple mechanisms, including blocking the respiratory chain of microorganisms [106], impairing membrane integrity [107], inhibiting microbial replication, transcription, and translation [108], regulating cytokine levels, and attenuating inflammatory responses [109]. AgNPs significantly enhance the antimicrobial effect when incorporated into composite systems such as curdlan–chitosan (CS) foams, which have demonstrated markedly improved wound regeneration in type II diabetic models [110]. Notably, recent strategies have addressed oxidative toxicity through the use of smart delivery platforms. A notable example includes glucose-responsive HA hydrogels embedded with tea polyphenol-stabilized AgNPs, which dynamically modulate antimicrobial activity in response to hyperglycemic conditions [111]. 

#### 3.1.2. AuNPs

Gold nanoparticles (AuNPs) are widely utilized in drug delivery and tissue regeneration [112]. They exert antimicrobial effects by forming cavities in microbial cell walls, interfering with ATP synthesis and DNA replication [113]. AuNPs also exhibit significant antioxidant activity, capable of scavenging free radicals and inhibiting the generation of ROS/RNS under hyperglycemic conditions, a critical feature in diabetic wound environments [114,115]. However, AuNPs alone may trigger hypersensitivity reactions, limiting their standalone use. Composite biomaterials have been developed to solve this problem. For instance, a Schiff base-crosslinked hydrogel incorporating Au–Pt alloy nanoparticles within oxidized HA/carboxymethyl chitosan (CMCS) networks significantly improved wound contraction in diabetic rat models by neutralizing oxidative stress and promoting angiogenesis without inducing detectable toxicity [116]. 

#### 3.1.3. CuNPs

Copper nanoparticles (CuNPs) exhibit broad-spectrum antimicrobial effects primarily by releasing Cu^2+^ ions, which disrupt microbial protein activity and induce cell death [117,118,119,120]. However, repeated application of copper oxides or copper salts on the wound surfaces may result in toxicity risks [121]. To address this, advanced delivery systems have been developed to ensure controlled and gradual release of copper ions. For example, calcium-alginate hydrogels loaded with CuNPs and deferoxamine (DFO) enable sustained Cu^2+^ ion release, reducing cytotoxicity while accelerating wound healing in diabetic mouse models [122]. A recent study introduced a copper-based metal-organic framework (MOF) loaded with taxifolin and demonstrated outstanding catalase-like activity [123].

#### 3.1.4. ZnONPs

Zinc oxide nanoparticles (ZnONPs) are considered superior to other NPs due to their high bioavailability and maximum first-pass metabolism [124]. They exhibit size-and dose-dependent antimicrobial activity through multiple mechanisms, including the induction of intracellular ROS, the release of Zn^2+^ ions, and adhesion to the cell membrane to disrupt cellular integrity [125,126]. Compared to Gram-positive bacteria strains, wound dressings containing ZnONPs demonstrate higher antimicrobial efficacy and longer maintenance time against Gram-negative bacteria [127]. Carbon fabrics loaded with ZnO nanoparticles (ZnONPs@CF) significantly enhanced wound contraction, with histopathological analysis revealing no detectable skin toxicity in diabetic rats [128]. Interestingly, another study has reported that pure ZnONPs exhibit superior antibacterial activity compared to hydrogels that incorporate ZnO, suggesting that direct application of ZnONPs may sometimes offer greater therapeutic benefit [129]. To further illustrate the properties and potential limitations of metallic NPs in diabetic wound management, a comparative overview is provided in Table 5.

### 3.2. Non-Metallic NPs

#### 3.2.1. SiO_2_ NPs

Silicon dioxide (SiO_2_), naturally occurring in biological systems as networked nanoaggregates, plays key roles in the formation of bone, cartilage, and connective tissue. Leveraging the excellent drug-loading capacity of silica, curcumin has been successfully loaded onto the surface of silica nanoparticles (SiO_2_ NPs), resulting in enhanced antimicrobial activity [130]. Furthermore, a silica–collagen I nanocomposite hydrogel has demonstrated sustained drug release capabilities and antimicrobial activity, facilitating wound healing in chronic diabetic wounds [131]. 

#### 3.2.2. Carbon NPs

Carbon NPs can be roughly classified into carbon dots, carbon nanotubes, and graphene/graphene oxide based on the structural configuration of carbon atoms [132]. These nanomaterials have demonstrated broad-spectrum antimicrobial activity against invasive bacteria, fungi, and viruses through inactivating bacterial enzymes, reducing oxidative damage to cellular components, and physically disrupting cell membranes [133,134]. Recent advancements have further enhanced the therapeutic potential of carbon-based nanomaterials in wound healing. For instance, Dai et al. [135] developed zinc single-atom nanozymes supported on carbon dots (Zn/C-dots), which exhibit potent ROS scavenging, antibacterial, and pro-angiogenic activities. The Zn/C-dots nanozymes, integrated into ROS-responsive hydrogels, significantly accelerated diabetic wound healing by reducing inflammation, promoting collagen deposition, and stimulating angiogenesis. 

## 4. The Molecular Mechanism of NPs with Natural Products on the Healing of Diabetic Wounds

### 4.1. Blood Glucose Control

The hyperglycemic microenvironment in diabetic wounds impedes the normal healing process; thus, lowering local blood glucose levels has emerged as a key therapeutic strategy. Nanomedicine-based delivery systems offer a promising approach by transporting enzymes or natural compounds to modulate glucose concentrations at the wound site. For example, glucose oxidase (GOx)-loaded nanoreactors can catalyze the oxidation of glucose to gluconic acid and hydrogen peroxide, enabling localized “starvation therapy” [3,136,137]. Gluconic acid acidifies the microenvironment and facilitates the release of drugs from pH-responsive nanoplatforms, while hydrogen peroxide has broad-spectrum antimicrobial effects that directly kill bacteria [138]. By consuming glucose, GOx-nanoenzymes not only improve the hyperglycemic microenvironment of the wound but also deprive microbes of essential nutrients, producing multidimensional therapeutic effects [139]. Additionally, some naturally occurring small molecules with hypoglycemic effects, such as sesamol, have shown promise. When incorporated into nanocarriers for controlled release, these agents may enhance systemic glucose metabolism, thereby indirectly supporting wound healing [37].

### 4.2. Infection Control

Persistent infections and biofilm formation often complicate chronic diabetic wounds. The incorporation of natural antimicrobial agents into nanomaterials enables sustained and adequate clearance of drug-resistant bacteria [140]. A berberine-loaded Spirulina platensis hydrogel (BBR@SP gel) eliminates biofilms via sustained ROS generation and downregulation of virulence factor [141]. A novel nanoVelcro dressing made from quercetin polymerization and crosslinking under alkaline catalysis effectively suppresses the growth of Gram-negative, Gram-positive, and multidrug-resistant bacteria in complex media (e.g., human plasma) through ultrastrong bacterial wrapping [142]. This dual physical-chemical antimicrobial mechanism significantly reduces wound bacterial burden and biofilm thickness on the wound surface. By controlling infection, immune activation triggered by endotoxins is reduced, leading to the inhibition of chronic inflammatory pathways and providing a clean microenvironment for tissue repair.

### 4.3. Regulation of Immunity

Persistent inflammation in diabetic wounds is closely associated with immune dysregulation, particularly the sustained activation of M1-type macrophages and prolonged elevation of pro-inflammatory cytokines [143]. NPs incorporating natural products have shown great promise in reshaping the immune microenvironment and promoting the transition from the inflammatory phase to tissue repair.

On one hand, macrophage phenotype reprogramming can be achieved via targeted delivery of pro- and anti-inflammatory factors. Among the signaling pathways regulating macrophage polarization, the STAT family of transcription factors orchestrates the determination of M1/M2 fate [144]. Nanomaterials can induce M2-type polarization by simultaneously suppressing STAT1 activity and activating STAT6, thereby promoting anti-inflammatory and pro-reparative responses [96]. An IL-33-loaded zeolitic imidazolate framework (ZIF) nanoplatform has been developed to address tissue regeneration disorders by enhancing M2 polarization and reversing the chronic pro-inflammatory state of diabetic wounds [145].

Alternatively, natural anti-inflammatory compounds, such as plant-derived polyphenols, directly modulate immune cells via nanodelivery systems to intervene in inflammatory signaling pathways. For instance, a berberine-loaded Spirulina platensis hydrogel (BBR@SP gel) has been shown to accelerate the healing of MRSA-infected diabetic wounds by suppressing inflammatory responses [141]. Similarly, CS-based hydrogels exert dual regulatory effects by upregulating anti-inflammatory cytokines such as IL-4 and TGF-β1 while simultaneously inhibiting IL-6 expression [91,92]. Furthermore, a GelMA/SFMA/MSN-RES/PDEVs nanocomposite system promotes angiogenesis and accelerates wound closure in diabetic mice by balancing the expression of TNF-α/iNOS and TGF-β1/Arg-1 [62]. The sesamol nanofiber membrane further contributes to wound healing by alleviating the inhibition of IL-6 and upregulating IL-10 expression, thereby stimulating keratinocyte proliferation [38].

### 4.4. Inducing Angiogenesis or Improving Local Microcirculation

Microangiopathy in diabetic tissues causes insufficient blood supply, local hypoxia, and nutrient deprivation in wounds. Therefore, promoting angiogenesis is a crucial therapeutic strategy to accelerate wound healing. The integration of nanomaterials with natural products has demonstrated the potential to stimulate neovascularization and enhance microcirculatory perfusion through multiple mechanisms. First, nanodelivery systems enable localized expression of vascular growth factors. For instance, gene-activated bilayer dermal equivalents incorporating nanosize complexes of Lipofectamine 2000 (Carlsbad, CA, USA) and plasmid DNA encoding VEGF into a collagen–chitosan scaffold/silicone membrane bilayer significantly upregulate VEGF expression in vitro, facilitating oriented collagen deposition and rapid re-epithelialization [146]. Second, pro-angiogenic trace elements such as copper can be delivered in a controlled manner. Copper binds stably to copper transporter protein 1 (CTR1), sustaining VEGFR2 signaling independently of Cu transporter pathways. The recycling of internalized CTR1 and VEGFR2 to the plasma membrane may trigger a copper uptake–lysyl oxidase (LOX) axis-mediated angiogenic response [147]. However, high concentrations of free Cu^2+^ are cytotoxic. To mitigate this, copper-based metal-organic framework nanoparticles (MOFs) incorporated into an antioxidant, thermoresponsive citrate-based hydrogel have been employed in diabetic mice to reduce ion toxicity while promoting angiogenesis and tissue repair [148]. Third, engineered living cells can serve as microbioreactors to supply pro-angiogenic factors continuously. A heparin–poloxamer thermoresponsive hydrogel containing living Lactococcus bacteria has been developed to synthesize and protect VEGF, enhancing endothelial cell migration, proliferation, and tube formation [149]. Fourth, natural product-based systems can exert indirect pro-angiogenic effects. The berberine-loaded Spirulina platensis hydrogel (BBR@SP gel) can accelerate wound healing in diabetic patients infected with MRSA by promoting angiogenesis [141]. Similarly, the combination of Aloe gel and olive oil promotes wound healing properties by upregulating markers of cell proliferation (Ki-67) and angiogenesis (CD34) [150]. Finally, natural compounds such as curcumin and resveratrol, known for their antioxidant and microcirculatory-enhancing properties, can boost nitric oxide (NO) production, thereby improving tissue oxygenation and supporting neovascularization. [28,46,51,62,64,70].

### 4.5. Removing Local Senescent Fibroblasts

Chronic difficult-to-heal wounds often exhibit an abnormal accumulation of senescent cells, especially senescent fibroblasts [151]. These cells secrete chemokines and inflammatory cytokines to remove senescent cells but also amplify the local inflammation [152]. The recent emergence of nanocarrier-based strategies for removing senescent cells has shown promise in diabetic wounds [153]. A representative approach utilizes nanomedicine delivery systems designed to target specific surface receptors expressed on senescent cells, thereby enabling selective elimination of these dysfunctional cells. A talabostat-modified poly-l-lysine/sodium alginate platform incorporating a PARP1 plasmid delivery system specifically targets the dipeptidyl peptidase 4 receptor, thus eliminating senescent fibroblasts, reducing senescence-associated secretory phenotypes, increasing the release of anti-inflammatory factors, speeding up collagen deposition and re-epithelialization, and intensely stimulating macrophage polarization towards M2 phenotype, which helps with tissue repair and the inflammatory response [154].

### 4.6. Promotion of Endothelial Cell Migration

Endothelial cell migration is critical for neovascularization and epithelial repair during wound healing. To promote the migration of vascular endothelial cells from the wound margins to the wound center, researchers have incorporated biophysical stimulation into natural materials. For example, Wang Xiao-Feng et al. developed a wound care system by combining chitosan–Vaseline® gauze with an electrical stimulation device, demonstrating that high-voltage monophasic pulsed current significantly accelerated diabetic wound healing through PI3K/Akt and ERK1/2-mediated activation of human umbilical vein endothelial cell proliferation and migration [155]. In addition, it has been shown that astragaloside IV enhances epithelial regeneration, endothelial cell migration, and lumen formation by upregulating key proteins involved in the SUMOylation pathway, including PCNA, Ras, HIF-1α, PPARγ, and VEGFR2 [57]. Moreover, astragaloside IV enhances epithelial regeneration, endothelial cell migration, and lumen formation through the SUMOylation pathway, involving proteins such as PCNA, Ras, HIF-1α, PPARγ, and VEGFR2.

**Figure 2:**
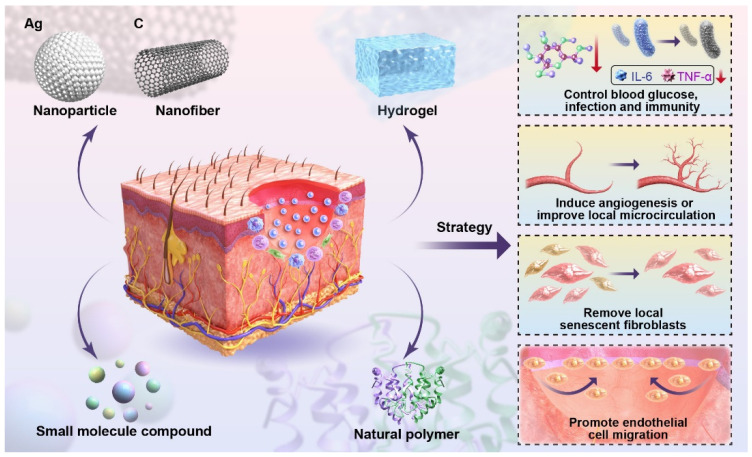
Schematic diagram of the molecular mechanisms of nanomaterials in treating the diabetic wound. This schematic illustrates application strategies of different biomaterials (NPs, nanofibers, HA, small molecule compounds, natural polymers) on the skin to achieve functional therapies for diabetic wounds, including controlling blood glucose, infection, and immunity; inducing angiogenesis; improving local microcirculation; removing local senescent fibroblasts; and promoting endothelial cell migration.

Figure 2 presents a schematic illustration of these integrated molecular mechanisms, providing a visual summary of how nanomaterials promote comprehensive tissue repair in diabetic wounds.

## 5. Advantages of Nanomedicine in the Healing of Diabetic Wounds

### 5.1. Safety of Medication Use

As previously discussed, various nanomaterials have markedly mitigated drug toxicity by enabling controlled and targeted release [116,122]. However, beyond the pharmacological properties of the drug itself, the local wound microenvironment plays a critical role in influencing therapeutic outcomes. Alkaline pH conditions in diabetic wounds are associated with enhanced bacterial colonization, progressive necrosis, and cellular degeneration, all of which exacerbate chronic inflammation and hinder healing [156]. Notably, the efficacy of specific innovative therapeutic systems, such as pH-responsive antimicrobial hydrogels embedded with nanofiber networks, exhibits optimal biofilm eradication and wound repair capacity, underscoring the importance of microenvironment modulation in diabetic wound care [157]

### 5.2. Multiple Functions Simultaneously

Nanomaterials are capable of integrating multiple therapeutic functions to achieve synergistic effects. For example, Zn^2+^-coordinated curcumin-based metal-organic frameworks incorporated within hierarchically structured PLLA micro/nanofibrous scaffolds significantly enhance curcumin bioavailability, stimulate skin cell adhesion and proliferation, trigger inflammation-responsive curcumin release while promoting collagen matrix reconstruction, neovascularization, and epithelial regeneration during wound healing [158]. Another multifunctional hydrogel prepared by enzymatic crosslinking of EGCG dimer-grafted HA and tyramine-grafted human-like collagen and integrating deferoxamine (DFO)-loaded mesoporous polydopamine NPs exhibits prominent enhancement of angiogenesis and induces macrophage polarization from the pro-inflammatory M1 phenotype to the reparative M2 phenotype, demonstrating anti-inflammatory, antibacterial, antioxidant, and hemostatic properties [159].

### 5.3. Advantageous Drug Carrier

Compared to conventional dressings, nanocarriers offer significant advantages by enabling the encapsulation, protection, and sustained release of therapeutic drugs to the wound surface, thereby prolonging the duration of pharmacological activity and accelerating the overall wound healing process [160]. In addition, nanomedicines significantly enhance the bioavailability and delivery efficiency of pharmaceutical agents. Owing to their nanoscale size, these systems facilitate the penetration of drugs through cellular membranes and their access to intracellular compartments, thereby improving targeting precision and therapeutic outcomes.

## 6. Challenges for Nanomedicines 

Today, plant extracts have demonstrated significant potential in promoting diabetic wound healing, but their poor aqueous solubility, rapid degradation, and low bioavailability substantially limit their therapeutic efficacy [29,65,161]. Fortunately, nanomedicine offers efficient solutions to these challenges through targeted drug delivery and controlled release [162]. These nanoformulations can also improve the pharmacological performance of bioactive compounds by increasing their surface area-to-volume ratio, which is particularly advantageous for the prolonged and complex process of wound healing. Nevertheless, translational challenges remain, including drug delivery rates, potential toxicity, and the unpredictable release profiles of natural compounds due to their multi-target mechanisms [163,164]. Additionally, the intricate manufacturing processes and high production costs of nanomaterials hinder their large-scale clinical translation. Thus, comprehensive safety evaluations and rigorous in vivo studies are essential to ensure the clinical applicability of these approaches [165,166]. The integration of nanomaterials with natural compounds should also consider their compatibility and safety within biological systems. For example, the use of silk fibroin nanofibers highlights the importance of biocompatibility and mechanical strength in designing effective wound-healing platforms. Notably, silk-based biomaterials, particularly those fabricated through electrospinning technology, have been demonstrated to accelerate wound closure and tissue regeneration in both preclinical and clinical studies. 

Additionally, drugs encapsulated within nanomedicine are inherently time-, dose-, and patient-specific. Therefore, future research should emphasize comprehensive in vivo evaluations to ensure the safe and effective application of natural nanomedicines in diabetic wound management. Artificial intelligence (AI)-driven innovations are transforming the development paradigm—reinforcement learning platforms trained on thousands of simulated wound healing models optimize the selection of optimal carriers and natural compounds tailored to individual patient conditions. Simultaneously, a machine learning model trained with more than 12 physicochemical parameters of the natural compounds (e.g., solubility, binding affinity) can predict the optimal drug release profile to maintain therapeutic concentrations within the wound microenvironment. Nanorobotics also holds great promise for diabetic wound care by overcoming key barriers such as biofilms, tissue exudates, and impaired vasculature, which hinder drug delivery [167]. To realize their clinical potential, future studies must focus on enhancing the biocompatibility of nanorobots, minimizing issues such as ROS generation and toxicity, and customizing treatment strategies to address patient-specific variables, including hormonal and enzymatic profiles. Continued advancement in natural product-nanotechnology integration will revolutionize diabetic wound care, enabling precise, multitargeted, and safe therapeutic solutions. 

## Figures and Tables

**Table 1:** Nanotechnology-based scaffold loaded with small-molecule compounds in Diabetic wounds healing.

Nanomaterial	Biomolecule or Drug	Mechanisms	Ref.
Polyvinyl alcohol (PVA)–sodium alginate (SA)–SF-based multifunctional nanofibrous scaffold	Asiaticoside	Enhanced keratinocyte proliferation and migration. Controlling infection.	[15]
Aloe vera or Hypericum perforatum oil-loaded nanofiber	Hypericum perforatum oil	Regulating immunity	[16]
	Aloe vera	Regulating immunity	
SF/glycyrrhizic acid/Zn hybrid hydrogel	Glycyrrhizic acid	Regulating immunity	[17]
SF–melanin-berberine composite hydrogel	Berberine and melanin	Regulating immunity	[18]
Chitosan (CS)/PVA/Zinc oxide (ZnO) nanofibrous membranes	-	Controlling infection	[19]
CS-PVA-ZnO–Curcumin electrospun nanofibers	ZnO and curcumin	Regulating immunity. Promoting endothelial cell migration.	[20]
Collagen-CS scaffold	Pioglitazone	Regulating immunity	[21]
Asiatic acid-embedded aligned porous poly (l-lactic acid) (PLLA) electrospun fibrous scaffold	Asiatic acid	Regulating immunity Inducing Angiogenesis	[22]
*Malva sylvestris*-neomycin sulfate nanofibers	*Malva sylvestris* extract and neomycin sulfate	Controlling infection	[23]
Three-layered Polycaprolactone (PCL)-collagen nanofibers	Melilotus officinalis extract	Regulating immunity Inducing angiogenesis	[24]
Gelatin/CS bilayer nanofibrous scaffolds	Curcumin and lithospermi radix extract	Regulating immunity Inducing angiogenesis	[25]
SF/poly-(l-lactide-co-caprolactone) (PLCL) nanofiber scaffolds	Huangbai Liniment	Regulating immunity	[26]
Injectable and microporous microgel–fiber granular hydrogel loaded with bioglass and siRNA	small interfering RNA (siRNA) and bioglass	Regulating immunity Inducing angiogenesis	[27]

**Table 5:** Metallic NPs in diabetic wound healing.

Material	Properties	Limitations	Ref.
AgNPs	Broad-spectrum antimicrobial activity. Anti-inflammatory via modulating cytokine production.	Potential cytotoxicity at high concentrations. Risks of silver accumulation in tissues.	[106,110,111]
AuNPs	Antioxidant and antimicrobial properties. Promoting angiogenesis and tissue regeneration.	Potential biocompatibility issues.	[112,113,114,115,116]
CuNPs	Antimicrobial effect.	Dose-dependent cytotoxicity.	[117,118,119,120,121,122]
ZnONPs	Effective antimicrobial activity.	Potential ROS-induced cytotoxicity. Formulation-dependent efficacy.	[124,125,126,127,128]

## Data Availability

The data presented in this study are available on request from the corresponding author.
